# Analysis of Plasma Cytokine and Chemokine Profiles in Patients with and without Tuberculosis by Liquid Array-Based Multiplexed Immunoassays

**DOI:** 10.1371/journal.pone.0148885

**Published:** 2016-02-16

**Authors:** Wenjing Xiong, Haiping Dong, Juanjuan Wang, Xiaoming Zou, Qian Wen, Wei Luo, Sudong Liu, Jianchun He, Shaoxi Cai, Li Ma

**Affiliations:** 1 Institute of Molecular Immunology, School of Biotechnology, Southern Medical University, Guangzhou 510515, China; 2 Department of Respiratory and Critical Care Medicine, Nanfang Hospital, Southern Medical University, Guangzhou 510515, China; 3 The First People’s Hospital of Kashi, Xinjiang 844000, China; University of Cape Town, SOUTH AFRICA

## Abstract

The aim of this study was to establish plasma cytokine/chemokine profiles in patients with 3 different presentations of active tuberculosis (TB), compared to the profiles observed in bacillus Calmette-Guérin (BCG)-vaccinated healthy individuals and patients with other pulmonary diseases (non-TB patients). To this end, plasma samples were collected from 151 TB patients including 68 pulmonary TB (PTB), 43 endobronchial TB, and 40 tuberculosis pleurisy (TP) patients, as well as 107 no-TB cases including 26 non-TB patients and 81 BCG-vaccinated healthy controls. A liquid array-based multiplexed immunoassay was used to screen plasma samples for 20 distinct cytokines and chemokines. Multinomial logistic regression was used to analyze associations between cytokines/chemokines and TB/non-TB patients. Compared to our findings with the no-TB donors, the median plasma levels of the proinflammatory cytokines/chemokines TNF-α, IL-6, IP-10, IFN-γ, and MIP-1β were significantly elevated in TB patients, suggesting their potential use as biomarkers for diagnosing TB patients. Further comparisons with healthy donors showed that only the median TNF-α plasma level was highly produced in the plasma of all 3 types of TB patients. Plasma IL-6 production was higher only in TP patients, while the plasma levels of IP-10, IFN-γ, and MIP-1β were markedly enhanced in both PTB and TP patients. Unexpectedly, among the above cytokines/chemokines, MIP-1β was also highly expressed in non-TB patients, compared with healthy donors. Our results suggested that TNF-α may be an ideal biomarker for diagnosing the 3 forms of TB presentation, while the other factors (IL-6, IP-10, MCP-1, and IFN-γ) can potentially facilitate differential diagnosis for the 3 TB presentation types. Further characterization of immune responses associated with different types of TB diseases will provide a basis for developing novel TB diagnostics.

## Introduction

*Mycobacterium tuberculosis* is a virulent bacterial pathogen that can persist in host macrophages by blocking phagolysosome functions [1]. This bacterium causes millions of deaths annually worldwide, especially in developing countries. Disease development and outcomes associated with tuberculosis (TB) are dependent on the ability of the host to elicit a potent cell-mediated immune response, resulting in macrophage activation. Cytokines, such as tumor necrosis factor-α (TNF-α), interferon-γ (IFN-γ), interleukin-2 (IL-2), and IL-10, mediate signaling among T cells, dendritic cells, macrophages, and other immune cells. These interactions can define the disease outcome, presentation, and/or disease severity [2, 3]. IFN-γ-release assays are commonly used to diagnose latent or active *M*. *tuberculosis* infections. These assays are designed to measure the levels of IFN-γ (using enzyme-linked immunosorbent assays [ELISAs] or enzyme-linked immunospot [ELISPOT] assays) after stimulating whole-blood samples with *M*. *tuberculosis*-specific antigens (e.g., ESAT-6, CFP10, and TB7.7). Although diagnosis of TB using the IFN-γ-release assay is more effective and sensitive than using the tuberculin skin test, it remains difficult to distinguish active from latent TB infections [4, 5].

Recently, cytokine profiles in TB patients have been studied to develop novel treatment and diagnostic approaches [6–9]. Assessing the levels of different cytokines and chemokines produced in active TB patients and healthy household contacts enabled detection of *M*. *tuberculosis* infection. Mihret *et al*. demonstrated that the levels of epidermal growth factor, CX3CL1, IFN-γ, IL-4, monocyte chemotactic protein 1 (MCP-1), and IFN-γ inducible protein (IP-10) were significantly higher in patients with active infection than in latently infected or treated TB patients, suggesting that such measurements could be used for diagnosing *M*. *tuberculosis* infection and monitoring the efficacy of anti-TB therapy [9].

Pulmonary tuberculosis (PTB) is the most common form of TB presentation [10]. Endobronchial tuberculosis (EBTB), which occurs in approximately 10%–40% in all TB patients [11], occurs when TB presents following infection of the bronchial tubes. In addition, approximately 25%–27% of TB patients develop extrapulmonary tuberculosis, in which TB presents outside of the pulmonary tissues [12]. One of the most common extrapulmonary TB diseases, tuberculosis pleurisy (TP) represents nearly 20% of extrapulmonary TB diseases [13] and is generally considered to be a reactivated form of *M*. *tuberculosis*. However, few *M*. *tuberculosis* organisms are detectable in pleural effusions, even in those from TP patients. The gold standard for diagnosing TP involves the examination of pleural biopsies, which is effective, but still highlights the need to identify more convenient and novel diagnostic biomarkers [14–16].

We found that the level of TNF-α was significantly enhanced in patients with all 3 forms of TB presentation, when compared to that observed in healthy individuals. The expression levels of TNF-α, IFN-γ, IP-10, and IL-6 may be used to discriminate TB patients from no-TB individuals, and examining the levels of IL-6 and MCP-1 may discriminate TP and PTB from other types of TB patients, respectively.

## Materials and Methods

### Study population

Two hundred and thirty-two patients were initially recruited from the Guangzhou Chest Hospital and the Nanfang Hospital. Patients with pregnancy and complications such as diabetes, autoimmunity diseases, and other chronic diseases including cancer and heart failure were excluded from this study.

Patients with persistent fever and cough lasting more than 3 weeks and receiving ineffective treatment against non-*M*. *tuberculosis* respiratory infections were screened for TB with chest X-rays, acid-fast smear examinations, and anti-TB treatments. Subjects with 1 or more positive results in the above examinations and without any other symptoms were diagnosed as pulmonary TB patients. On this basis, patients with exudative pleural effusion were suspected as having TP and were further evaluated by performing pleural fluid adenosine deaminase (ADA) assays, tissue biopsies, and pathological examinations. EBTB was confirmed by fiber-optic bronchoscopy, chest computed tomography, bronchial washing fluid culture, or bronchial biopsy.

Infection with pathogens other than *M*. *tuberculosis*, including hepatitis B (HBV), hepatitis C, and human immunodeficiency virus (HIV), was also examined. Specifically, the blood IgG-HIV level was examined with an ELISA to identify HIV-positive individuals, and CD4^+^ T cell numbers were determined by flow cytometry. Patients who had received TB treatment for over 1 week or were pregnant were also excluded from this study. Thus, of the 232 patients initially recruited for this study, 18 patients with 2 or more types of TB presentations, 18 patients with coinfections of *M*. *tuberculosis* and other pathogens, 9 patients with lymphatic TB infections, and 36 patients with pulmonary lesions in addition to TB were excluded. Finally, 151 patients were enrolled in the study.

In addition, 81 BCG-vaccinated healthy donors were enrolled in the study as random control subjects from Southern Medical University Hospital and Nanfang Hospital; 26 non-TB patients were recruited from Guangzhou Chest Hospital and Nanfang Hospital as controls. Healthy donors had no history of contact with TB patients, and none of them had been ill during the week before sample collection. Moreover, all healthy donors and non-TB patients had a purified protein derivative (PPD)-skin test result of less than 9 mm. Control subjects were tested for latent TB infection by the antigen-specific IFN-γ assay T-SPOT.TB [4]. The non-TB patients included 6 with pulmonary disease and hepatitis B virus infection, 12 with pulmonary disease not caused by *M*. *tuberculosis* infection, 6 with *Mycoplasma pneumonia*, and 2 with lung cancer.

### Ethics statement

This study was conducted in accordance with the Declaration of Helsinki. All participants provided consent prior to participation. Consent from guardians on the behalf of minors enrolled in this study was also obtained. Verbal (not written) consent was obtained, due to the brief time interval before diagnosis as pulmonary tuberculosis and acceptance of drug therapy. After we clearly introduced and explained to the patients how our research was to be performed, blood samples were acquired immediately from the patients after they agreed. In addition, our study and consent procedure were approved by the Ethics Committee at Southern Medical University.

### Plasma samples

Peripheral blood samples (3 ml) from all patients and healthy control subjects were collected into EDTA and centrifuged for 10 min at 1000 × *g*. Plasma samples were collected and stored at −80°C until use. Frozen plasma samples were thawed, mixed by vortexing, and centrifuged for 10 min at 10,000 × *g* to remove particulates before the cytokine/chemokine assays were performed.

### Stimulation of peripheral blood mononuclear cells (PBMCs) with early-secreted antigenic target-6 (ESAT-6)

PBMCs isolated from healthy donors were treated with the 6-kDa ESAT-6 protein, which is secreted by *M*. *tuberculosis* and is absent from BCG. The production levels of cytokines/chemokines were compared between ESAT-6-stimulated and unstimulated cells. Initially, PBMCs were harvested from the peripheral blood of healthy donors. Briefly, 3 ml of peripheral blood was layered onto Ficoll-Hypaque (Shanghai Second Chemistry Factory, Shanghai, China). Next, PBMCs (5 × 10^5^/200 μl) were added to each well of a 96-well plate containing RPMI-1640 medium (Hyclone Ltd., Logan, UT, USA) with 10% fetal bovine serum (FBS; Hyclone Ltd.). After the addition of 10 μg/ml ESAT-6 or its solvent (RPMI-1640 medium), PBMCs were incubated at 37°C in 5% CO_2_ for 48 h. Supernatants were collected and stored at −80°C until use.

### Multiplex cytokine assay

The Multiplex Map Kit (Merck Millipore, Darmstadt, Germany) was used to quantify 20 different human cytokines and chemokines (divided into 4 main categories; Table 1), in accordance with the manufacturer’s instructions. Briefly, after pre-wetting the plates, 25 μl of matrix solution and 25 μl of Standard or Control reagents were added were added to the standard or control wells, respectively. Twenty-five microliters of assay buffer and 25 μl of serum matrix were added to the background well, and 25 μl assay buffer and 25 μl sample (dilution 1:1) were added to the sample wells. Twenty-five microliters of pre-combined beads were added to each well. After an overnight incubation at 4°C, the plates were washed twice, 25 μl of detection antibody was added to each well, and the plates were incubated for 1 h on a plate shaker. Twenty-five microliters of streptavidin-phycoerythrin was then added to each well, and the plates were shaken for 30 min at room temperature. Finally, the plates were washed 3 times, and 150 μl of sheath fluid was added to each well. Plates were read with a Luminex instrument (Luminex 200, Austin Luminex, USA). Data were analyzed using MILLIPLEX Analyst 5.1 software (Merck Millipore Darmstadt, Germany), in accordance with the manufacturer’s instructions. A standard curve for each cytokine was generated by mixing known concentrations of recombinant human cytokines.

**Table 1:** Classification of detective cytokines and chemokines.

Cytokines/ Chemokines	
**pro-inflammatory cytokines**	interferon gamma (IFN-γ), Tumor necrosis factor A (TNF-α), IL-12p40, IL-12p70, IL-1β, IL-1α, IL-17A, IL-6 and IL-8
**anti-inflammatory cytokines**	IL-10, and IL-13
**chemokines**	IFN-γ inducible protein (IP-10/CXCL-10), monocyte chemotactic protein 3(MCP-3/CCL7), monocyte chemotactic protein 1 (MCP-1/CCL2), monocyte inflammatory protein 1 beta (MIP-1β/CCL4) and Fractalkine (CX3CL1,FKN)
**Growth factors**	granulocyte macrophage colony stimulating factor (GM-CSF), vascular endothelial growth factor (VEGF), IL-2 and IL-5

### Statistical analyses

GraphPad Prism 5 was used to generate plots, and SPSS 17.0 was used to perform statistical analyses. The independent-sample T test was used to identify differences in cytokine-production levels between TB patients and no-TB donors. Differences in cytokine expression among the 3 types of TB patients, healthy donors, and non-TB patients were compared by one-way analysis of variance (ANOVA). Multiple comparisons were performed using the least significant difference (LSD) post-hoc test when the variance between samples was equal, or Dunnett’s T3 test when the variances were not equal. Comparison of ESAT-6-stimulated and unstimulated cells was performed using a paired-sample *t* test. Multinomial logistic regression was used to estimate the association of cytokine levels between patient groups. Odds ratios (ORs) and the corresponding 95% confidence intervals (CIs) are reported. Data are expressed as the mean ± standard error of the mean (SEM). *P* values < .05 were considered statistically significant.

## Results

### Population demographics

One hundred and fifty-one subjects with active TB were enrolled in this study from February 2013 to January 2015. Active TB patients included those diagnosed with EBTB (*n* = 43), TP (*n* = 40), or PTB (*n* = 68). No-TB cases, including 81 healthy donors and 26 non-TB patients, were also recruited (Table 2). All patients accepted systemic examination.

**Table 2:** Patient characteristics.

Baseline characteristics	BHD	PTB	EBTB	TP	non-TB
**number**	81	68	43	40	26
**Sex ratio (male/female)**	42/39	53/15	15/28	32/8	19/7
**Age range**	22–37	15–71	14–76	16–62	20–62
**Age mean ± SEM**	22.15±0.24	32.59±1.72	31.65±2.19	29.23±1.81	37.11±2.12
**Smear grade**					
+	-	37	26	23	0
≧++	-	28	16	11	0
unknown	-	3	1	6	0
**Number of cavities on chest X-ray**					
0	81	30	27	25	20
1	0	27	13	8	2
≧2	0	6	3	3	-
unknown	0	5	0	4	4
**PPD (mm)**					
< 9 mm	81	0	0	0	26
9 mm < PPD < 15 mm	0	46	30	24	0
PPD ≧ 15 mm	0	22	13	16	0
**T.SPOT.TB**					
negative/positive	70/0	2/55	0/40	0/35	26/0
unknown	11	11	3	5	0
**ADA**					
negative/positive	-	-	-	5/24	-
unknown				11	-

BHD, BCG-vaccinated healthy donors; PTB, pulmonary tuberculosis; EBTB, endobronchial tuberculosis; TP, tuberculosis pleurisy.

### Analysis of plasma cytokine and chemokine levels in TB and no-TB cases

**Fig 1:**
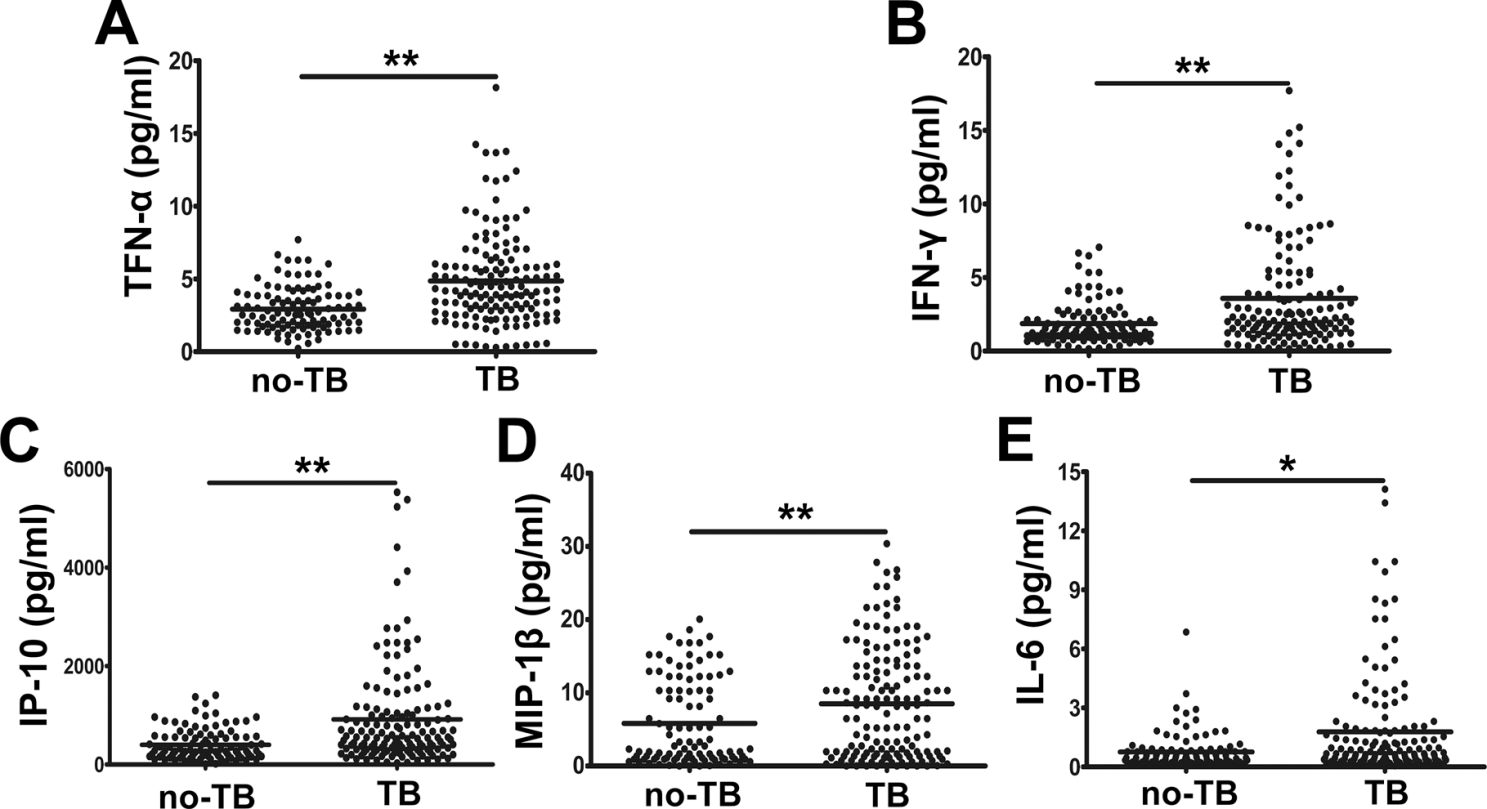
Representative plasma cytokine/chemokine expression levels in TB patients (*n* = 151) and no-TB donors (*n* = 107). (A) TNF-α, (B) IFN-γ, (C) IP-10/CXCL-10, (D) MIP-1β, and (E) IL-6 levels. *P < 0.05, **P < 0.001. *P*: *P* value. Independent-sample T testing was performed to compare the results from TB patients with those of no-TB cases.

First, we examined 20 cytokines/chemokines using Luminex technology. Plasma samples were obtained from 151 active TB patients and 107 no-TB cases (including 81 healthy donors and 26 non-TB patients). We then compared the expression of cytokine/chemokine levels between these 2 groups by performing an independent-sample T test. We found that among the 20 cytokines/chemokines examined, the levels of proinflammatory cytokines (TNF-α, IFN-γ, and IL-6) and chemokines (IP-10 and MIP-1β) in TB patients were significantly higher than those measured in no-TB cases (*P* < 0.05), when healthy donors and non-TB patients were regarded as one category (Fig 1). In contrast, no significant differences in the levels of other cytokines/chemokines were observed between the TB patients and no-TB cases.

### Variance in cytokine/chemokine expression levels with disease presentation

**Fig 2:**
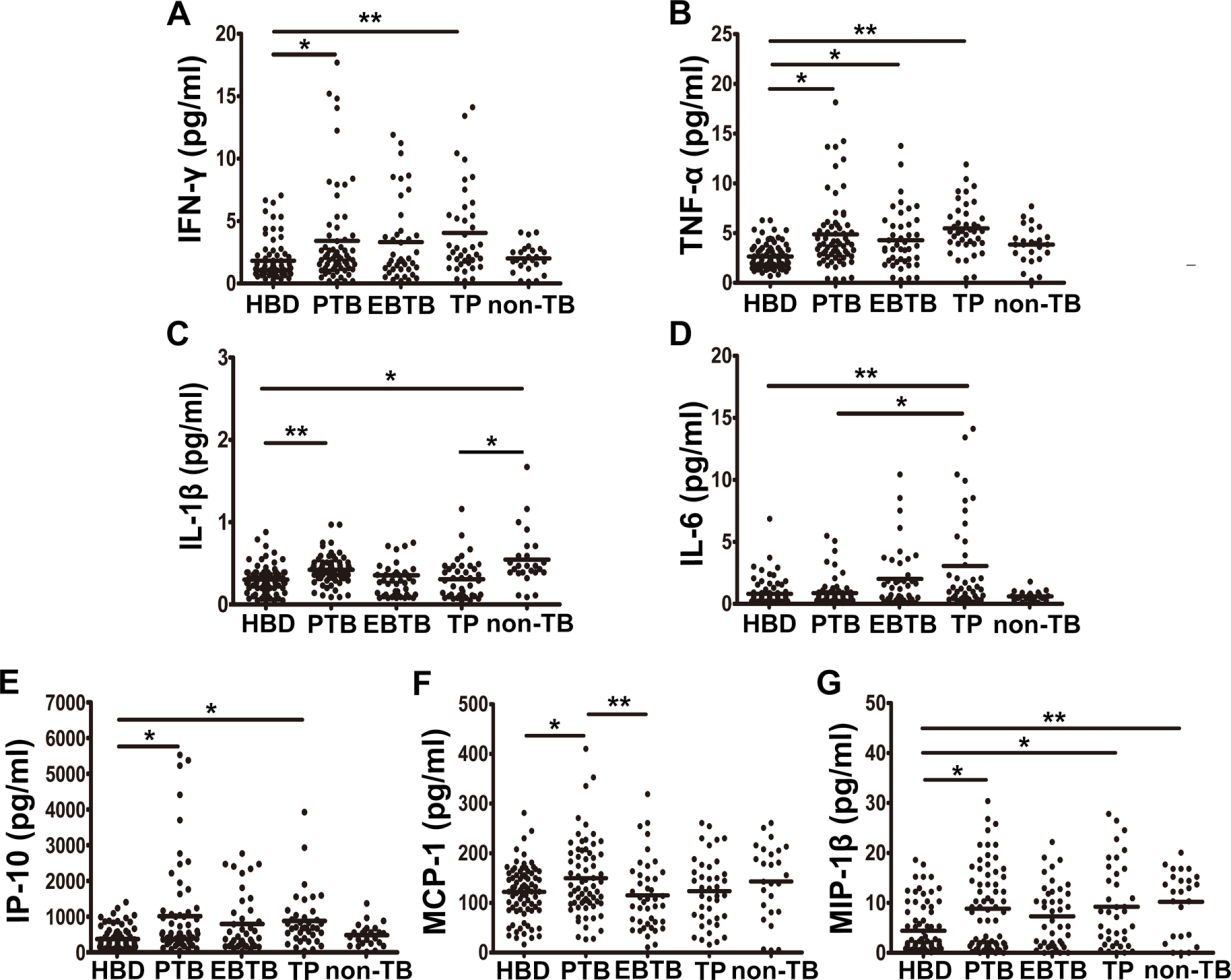
Cytokine/chemokine profiles were established for patients with 3 distinct presentations of TB, for BCG-vaccinated healthy donors (BHD), and for non-TB patients. The levels of (A) IFN-γ, (B) TNF-α, (C) IL-1β, (D) IL-6, (E) IP-10/CXCL-10, (F) MCP-1, and (G) MIP-1β are shown. *P < 0.05, **P < 0.001. *P*: *P* value; ANOVA, analysis of variance. When the variance between samples was equal, least significant difference (LSD) post-hoc tests were performed. Dunnett’s T3 test was used when the variance was not equal.

Second, the plasma levels of cytokines/chemokines for the 151 active TB patients and 81 healthy controls were compared to study whether expression of the above biomarkers formed unique profiles for each TB type. A group of non-TB patients was used as an additional control. Compared to the cytokine/chemokine-production levels observed in the no-TB controls, the median levels of 5 cytokines/chemokines (IFN-γ, TNF-α, IP-10, IL-1β, and MIP-1β) were significantly elevated in the plasma of PTB patients. The plasma MCP-1 levels were significantly higher in PTB patients than in EBTB patients, with whom only TNF-α was significantly increased. The plasma IFN-γ, TNF-α, IP-10, and MIP-1β levels were also higher in TP patients than the corresponding levels observed in healthy control subjects, while IL-6 production was markedly higher in TP patients than in PTB patients. Unexpectedly, the median plasma levels of IL-1β and MIP-1β in non-TB patients were also significantly higher than observed in healthy donors and TP patients (*P* < 0.05, Fig 2). No other cytokines or chemokines showed significant differences in expression levels between patients and healthy controls.

### Association of cytokine/chemokine expression levels with TB/no-TB donors

Next, associations between different biomarkers and different patient groups were examined by multivariable regression analysis. Using the no-TB cases as a reference category, with adjustment for demographic variables including gender and age, we found that the TNF-α level was 1.46 times more likely to be elevated in TB patients (OR 1.459, 95% CI 1.169–1.821), whereas the IP-10 level was approximately 1 time more likely to be elevated in TB patients (OR 1.00, 95% CI 1.000–1.002).

### Association between cytokine/chemokine levels and TB/non-TB patients

The data from all non-TB and TB patients were analyzed by multivariable regression analysis, after adjustment for sex and age. Using healthy controls as a reference category, IL-1β levels were 10 times more likely to be elevated in PTB patients, but 65 times more likely to be elevated in non-TB patients. Our analysis also showed that IP-10 was more associated with EBTB than with the other TB types, TNF-α was more associated with PTB and TP, and IL-6 was likely to be related to both TP and PTB (Table 3).

**Table 3:** Association of patient cytokine expression profiles and disease.

Independent variables	Type							
	PTB		EBTB		TP		Non-TB	
	OR (95% CI)	*P*	OR (95%CI)	*P*	OR(95%CI)	*P*	OR (95%CI)	*P*
**IL-1β**	10.71(1.25–92.08)	0.031	0.715(0.05–10.81)	0.809	0.601(0.03–11.71)	0.736	65.49(5.49–781.685)	0.001
**TNF-α**	1.49(1.11–1.99)	0.008	1.08(0.77–1.52)	0.645	1.518(1.103–2.09)	0.01	1.44 (0.979–2.114)	0.064
**IP-10**	1.00 (1.00–1.02)	0.07	1.002(1.00–1.007)	0.008	1.002(1.00–1.003)	0.013	1.00(0.998–1.002)	0.768
**IL-6**	1.07(0.73–1.58)	0.71	1.418(1.01–1.99)	0.045	1.504(1.067–2.12)	0.02	0.496(0.14–1.756)	0.277

*P*, *P*-value; OR, odds ratio; CI, confidence interval. Forward stepwise multivariable regression analyses were performed with adjustment for demographic variables (gender and age). Unstandardized regression coefficients. BHD, BCG-Vaccinated healthy donors; PTB, pulmonary tuberculosis; EBTB, endobronchial tuberculosis; TP, tuberculosis pleurisy.

### Biomarker expression profiles with ESAT-6 stimulation

**Fig 3:**
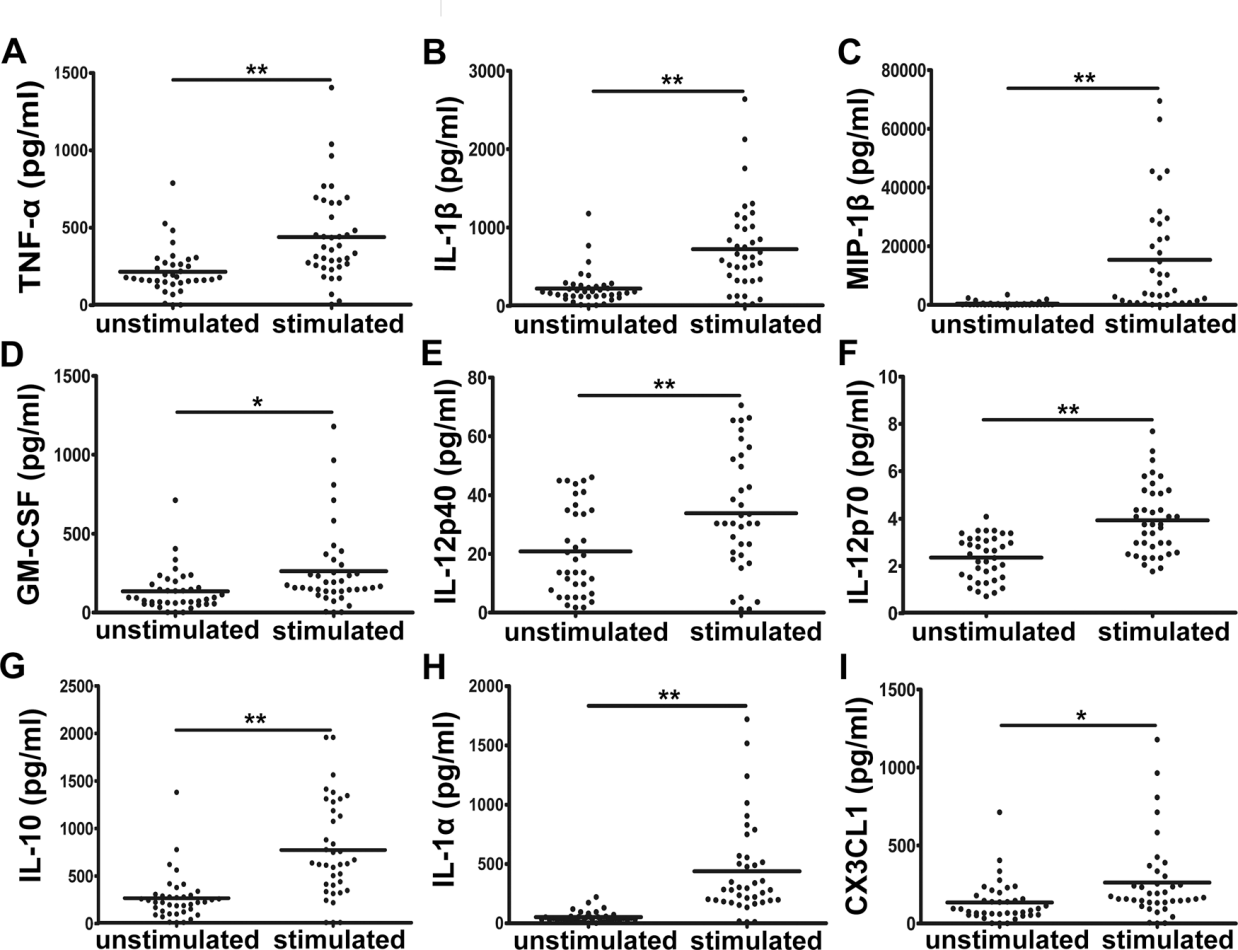
Cytokine/chemokine levels in PBMCs from BCG-vaccinated healthy donors (BHD), with or without ESAT-6 stimulation. The levels of (A) TNF-α, (B) IL-1β, (C) MIP-1β, (D) GM-CSF, (E) IL-12p40, (F) IL-12p70, (G) IL-10, (H) IL-1α, and (I) CX3CL1 are shown. *P < 0.05, **P < 0.001. *P*: *P* value. An independent-sample T test was performed.

ESAT-6 was used to stimulate the PBMCs of healthy donors to explore whether a unique *M*. *tuberculosis* antigen can activate the immunity of BCG-vaccinated healthy donors. The supernatants were screened for the presence of cytokines and chemokines, and the levels of these factors were compared to those detected from unstimulated cells. Consistent with the results observed in TB patients, the levels of IL-1β, TNF-α, MIP-1β, and IP-10 significantly differed in ESAT-6–stimulated PBMCs, compared to those levels in unstimulated cells (*P* < 0.05, Fig 3). In contrast, plasma CX3CL1 levels decreased after ESAT-6 stimulation (*P* < 0.05, Fig 3I), suggesting that *M*. *tuberculosis* antigens other than ESAT-6 may be responsible for the elevated plasma CX3CL1 concentrations in TB patients. In contrast, the levels of IL-12p40, IL-12p70, IL-10, and IL-1α significantly increased in supernatants collected from ESAT-6-stimulated cells, which was not observed with samples from the TB patients (*P* < 0.05, Fig 3).

## Discussion

Few studies have compared the cytokine profiles of patients with different TB presentations or have included patients with pulmonary conditions other than TB as controls. Using Luminex technology, we established the concentrations of different cytokines/chemokines in plasma samples from PTB, EBTB, TP, non-TB patients, and healthy controls from southern China. Associations between cytokine/chemokine expression levels and disease presentation were analyzed by multinomial logistic regression [17]. To verify that the observed changes in samples from TB patients resulted from *M*. *tuberculosis* infection, the *M*. *tuberculosis*-specific antigen ESAT-6, which is not expressed by the BCG vaccine strain, was used to stimulate PBMCs collected from healthy donors. The results were compared to those obtained with unstimulated cells. We found that: 1) Significantly higher plasma concentrations of TNF-α, IL-6, IFN-γ, IP-10, MCP-1, and MIP-1β were present in TB patients compared to the corresponding levels measured in no-TB individuals. 2) Compared with healthy donors, the plasma levels of TNF-α were significantly higher in all 3 types of TB patients; IL-6 showed elevated expression in TP patients alone; the plasma levels of MCP-1 and IL-1β were only increased in PTB patients; and IP-10, MIP-1β, and IFN-γ levels increased markedly in PTB and TP patients. 3) Among the cytokines/chemokines studied, TNF-α, IP-10, IL-6, and IL-1β were associated with 3 TB patients, but IL-1β was the most closely associated chemokine/cytokine with PTB among the TB types studied. However, the association of IL-1β levels with TB presentation was less apparent when compared to the association with non-TB patients. 4) Nearly 9 cytokines/chemokines were secreted by healthy donors’ PBMCs stimulated by ESAT-6, but not including IFN-γ, consistent with our T-SPOT.TB test results.

As a key proinflammatory cytokine, TNF-α plays important roles in resistance to infection and cancer [18], but exerts pleiotropic and complex functions in the context of TB. TNF-α can promote the phagocytosis of macrophages and the intracellular killing of *M*. *tuberculosis*; however, at excessive levels, TNF-α is associated with increased pathology [19]. Accordingly, perturbations of TNF-α expression can markedly affect the course of disease [20]. Some data have suggested that after stimulating CD4^+^ T cells with ESAT-6, the resulting high TNF-α levels can distinguish active TB patients from latent TB patients [21]. In agreement, we observed significantly higher TNF-α levels in all TB patients when compared to no-TB cases or only healthy donors, and TNF-α levels were more closely associated with PTB and TP patients when analyzed by multinomial logistic regression. Collectively, these observations suggested that the plasma level of TNF-α (without antigen stimulation) may serve as a biomarker to distinguish TB-positive from TB-negative individuals.

IFN-γ and IL-6 are important mediators of cellular immune responses. IFN-γ is mainly produced by activated Th1 cells, which leads to macrophage activation, killing of intracellular *M*. *tuberculosis*, and enhanced expression of MHC II molecules to promote antigen recognition [22, 23]. Various studies have been conducted to measure IFN-γ levels after stimulation of *M*. *tuberculosis*-specific antigens for diagnosing TB [24], and the IFN-γ level can distinguish active TB patients from latent TB patients [9]. Previously, we found that IFN-γ expression levels significantly increased in PTB and TP patients, suggesting that plasma IFN-γ levels of can also be used to discriminate TB patients from no-TB individuals. In addition, in BCG-vaccinated healthy donors’ PBMCs that were stimulated with ESAT-6, the IFN-γ level did not increase, suggesting that the healthy donors recruited for this study did not have latent TB. IL-6 serves pleiotropic roles in inflammation and immune responses and is essential for effective host immune responses after *M*. *tuberculosis* infection [25]. Some investigators have suggested that high IL-6 levels are associated with non-cavitary tuberculosis and can be regarded as a potent biomarker of *M*. *tuberculosis* infection [25–27]. Our results showed that IL-6 expression levels were significantly increased in TP patients and even higher than observed in PTB patients. Analysis of the association between cytokine/chemokine production levels and TB disease presentations demonstrated that IL-6 was closely associated with EBTB and TP patients. Thus, we suggest that IL-6 can serve as a valuable biomarker for distinguishing TP patients from healthy individuals and other types of TB patients.

Several chemokines, such as IP-10, MCP-1, and MIP-1β, have the potential to recruit and activate leukocytes in response to *M*. *tuberculosis* infection [28, 29]. Chemokines can mediate protective anti-TB immune responses by inducing inflammation; however, the overwhelming lung inflammation induced by chemokines can also drive lung pathology, including cavitation and tissue destruction [29]. High chemokine levels could promote *M*. *tuberculosis* proliferation or alter anti-*M*. *tuberculosis* immune responses, resulting in higher bacterial burdens [30]. In response to *M*. *tuberculosis* infection, IP-10 and MIP-1β primarily possess effector functions in the lungs [29, 31]. MCP-1, which is produced by pulmonary alveolar epithelial cells, mediates inflammation by regulating the migration and recruitment of monocytes in response to *M*. *tuberculosis* infection [32]. We found that IP-10 levels in PTB and TP patients were significantly higher than those levels in healthy donors, whereas the MCP-1 levels in PTB patients were significantly higher than those levels in EBTB patients or healthy donors. These data suggest that MCP-1 can potentially serve as a secondary biomarker for discriminating patients with PTB from other types of TB patients. MIP-1β levels were markedly increased in PTB and TP patients, but not in EBTB patients. In addition, plasma MIP-1β levels were significantly higher in non-TB patients than in healthy donors, but not significantly different among TB patients with different presentation types. The underlying reasons for the differential expression of these 3 chemokines, which were all secreted by macrophages and function in the lungs, merit further study. These findings are consistent with the extensive roles of these chemokines in the pathogenesis of both infectious and noninfectious diseases [33, 34].

IL-1β is a vital proinflammatory cytokine that mediates immune functions and participates in the resistance to mycobacterial infections during the early stages of infection [35]. However, previous data have suggested that IL-1β release can also have detrimental effects [36]. For example, excessive increases in IL-1β levels or decreased concentrations of the IL-1R antagonist may lead to increased lung cavity presentation in PTB [37]. IL-1β has primarily been associated with acute and chronic inflammation, and blocking IL-1β is a classic therapeutic method for controlling many auto-inflammatory diseases [38]. Compared to that of healthy donors, we found that the IL-1β expression level was significantly higher in PTB patients, but not in EBTB or TP patients. One possible explanation for this finding is that the IL-1β levels in PTB *vs*. TP patients are easier to measure in the plasma, compared with pleural effusions, respectively [39]. Although previous findings have demonstrated higher IL-1β expression levels occur in TB patients, few studies have included a comparison with non-TB patients. As an important pro-inflammatory cytokine, IL-1β is also expressed highly in the plasma of HBV-infected and *Mycoplasma*-infected individuals [40–42]. Some authors have proposed that IL-1β levels are elevated in and associated with lung cancer [42, 43]. Considering that these results hinted that IL-1β production may be indicative of the host inflammatory condition, when compared to patients with other diseases (especially autoimmune diseases) [38], it is reasonable to expect that plasma IL-1β levels would be also higher in non-TB patients. When multinomial logistic regression was used to estimate the association between cytokine/chemokine levels and the TB presentation type, IL-1β was >10 times more likely to be present at elevated levels in PTB patients compared to patients with other TB presentations. These results indicated that IL-1β expression was not uniquely increased in TB patients, similar with the findings of Djoba Siawaya *et al*. [44]. The low levels of IL-1β supported the observed absence of a highly active immune response in the TB patients participating in this study.

The BCG vaccine strain is different from *M*. *tuberculosis* strains in several ways, including differences in antigen profiles (e.g., ESAT-6, CFP10, and TB7.7 are expressed by *M*. *tuberculosis*, but not by BCG). The BCG vaccine is administered to essentially all children in China as part of the expanded immunization program. Thus, nearly everyone in China has received this vaccine. To explore whether the immunity of BCG-vaccinated healthy donors can be activated by a single unique antigen of *M*. *tuberculosis*, changes in cytokine/chemokine expression profiles were analyzed in ESAT-6-stimulated PBMCs isolated from healthy donors. Following stimulation, IL-1β levels were significantly elevated. The levels of TNF-α, IL-1β, MIP-1β, GM-CSF, IL-12p40, IL-12p70, IL-10, IL-1α, and CX3CL1 were also different in ESAT-6–stimulated PBMCs, compared to unstimulated cells, although IFN-γ production was not different. IFN-γ is secreted by memory T cells in response to *M*. *tuberculosis* infection only in individuals with related memory T cells. Based on this consideration, we confirmed that the BCG-vaccinated healthy donors were not latent TB patients. However, other cytokines/chemokines were detected in PBMCs from the ESAT-6-stimulated group. The reasons underlying the activation of PBMCs from healthy donors by ESAT-6 may include: 1) PBMCs include both innate immune cells and adaptive immune cells. During a 48-h stimulation, several kinds of innate immune cells may serve as the major sources of cytokines/chemokines, such as dendritic cells, macrophages, T cells, and natural killer cells, among others. 2) Owing to the ubiquitous heterologous immunity reported by Clute *et al*. [23] and Shen *et al*. [22] and also observed in our lab, as well as the phenomenon that T cells cross-react with peptide epitopes encoded by related or even unrelated pathogens, T cells in healthy donors may produce cytokines upon ESAT-6 stimulation due to a previous encounter with other pathogens.

This study has some limitations. For example, we compared results from TB patients with those of non-TB patients and sought to identify a specific biomarker(s) for discriminating TB patients from those with other pulmonary diseases. However, owing to the similar immune responses occurring with pulmonary lesions, we could not identify any cytokines/chemokines with higher expression in TB patients than in non-TB patients. To overcome this limitation, more patients with pulmonary diseases not caused by *M*. *tuberculosis* infection and more types of TB patients need to be enrolled. It is a great challenge to confirm the specificity of biomarkers for different TB presentation types and other pulmonary diseases. Chegou *et al*. reviewed several kinds of cytokines/chemokines as candidate biomarkers for discriminating active TB from latent TB and highlighted the need for further confirmation with larger clinical sample sizes [45].

In conclusion, few studies have directly measured plasma cytokine profile levels without stimulating PBMCs from patients with different TB presentation types, or have included patients with pulmonary conditions other than TB as controls. By high-throughput Luminex technology, we found that the plasma levels of IL-6, IFN-γ, TNF-α, IP-10, and MCP-1 may be used to discriminate TB patients from no-TB individuals. Furthermore, measuring the plasma levels of IL-6 and MCP-1 may serve as a novel secondary approach for diagnosing TP and PTB patients. The IL-1β levels were higher in, and more significantly associated with, non-TB patients than TB patients. These data suggest that IL-1β, as a proinflammatory cytokine, may be detectable in many diseases; therefore, IL-1β cannot serve as a specific biomarker for TB patients. Our results represent a step towards distinguishing between patients with TB and those with other pulmonary diseases. Additional confirmation in studies including greater numbers of patients diagnosed with non-TB pulmonary diseases is needed to determine the specificity of the identified markers in *M*. *tuberculosis* infection. These results may contribute to new cytokine-based diagnostics and treatment strategies for TB, as well as provide novel insights into the nature of anti-*M*. *tuberculosis* immune responses.
